# Role of miR-16, 146a, 19b and 720 gene expressions in the pathogenesis and diagnosis of vitiligo

**DOI:** 10.1038/s41598-024-83489-y

**Published:** 2025-01-06

**Authors:** Olfat G. Shaker, Talal A. Abd elrahim, Somia Azzam, Mai Mohamed El-Zook, Nesreen M. Aboraia

**Affiliations:** 1 Department of Medical Biochemistry and Molecular Biology, Faculty of Medicine, Cairo University, Cairo, Egypt; 2 Department of Dermatology, Faculty of Medicine, Fayoum University, Fayoum, Egypt

**Keywords:** Vitiligo, miRNAs, VASI, VIDA, Biochemistry, Biomarkers, Skin diseases

## Abstract

Vitiligo is a common long-term depigmenting skin disorder that is characterized by patches of skin losing their pigment. To evaluate serum/tissue levels of miR-16, 146a, 19b and 720 in vitiligo patients and healthy controls, also analyzing the correlations between all biomarkers to indicate whether those can be used to early diagnose vitiligo patients. Forty-subjects were included, divided into two equal groups, 20 healthy matched individuals and 20 vitiligo patients. For all groups a 5 mL venous blood sample for serum isolation was taken and analyzed for the serum level of miRNAs. For tissue analysis, 3 mm biopsy was taken. For all patients Vitiligo area scoring index (VASI), vitiligo disease activity (VIDA), disease duration and extent percentages were calculated. No significant difference was observed in age and sex ratio among the two groups (p > 0.05). Serum, expression levels for miR-16, 146a, and 19b were overexpressed in vitiligo patients as compared to healthy controls with p-value 0.000 for all biomarkers. While miR-720 was reported low in vitiligo patients compared to controls (p = 0.000). For tissue samples, miR-16, 146a, 19b were overexpressed in vitiligo patients with p-values 0.000, 0.000 and < 0.001 respectively, while for the expression level of miR-720 in tissue, the level was low compared to controls (p = 0.000). There are positive correlations between VASI and miR-16, 146a in serum and miR-146a in tissue. Also positive correlations between disease extent and both miR-16 and miR-146a in serum and in tissue was found. A negative correlation between VIDA and miR-720 in serum was found. Various correlations between the selected miRNAs were reported. Based upon the expression levels of miR-16, 146a, 19b and 720 in both serum and tissue, these biomarkers can be used as early indicators for vitiligo.

## Introduction

Vitiligo is an acquired chronic autoimmune asymptomatic pigmentary disorder that results in loss of functional melanocytes, causing white spots on the skin. It is mainly caused by the different factors including genetic, immune abnormalities, and psycho-psychological factors^1,2^.

Based on previous studies, genome‐wide microRNA (miRNA; miR) studies have been extensively used to elucidate the genetic and immune‐correlated pathogenesis of various skin diseases including vitiligo. miRNAs act as regulators of gene expression and regulate most of cellular processes including cell proliferation, differentiation, development and immune responses. They regulate immune cell development and are involved in autoimmune development^2–5^.

miR-16 is a regulator of immune-mediated tissue repair^6^. miR‐146 has a positive role in the control of the natural immune activity of monocytes/macrophages. The expression of miR146a/b is increased in response to cytokines, which then leads to the reduction of inflammatory responses by effecting multiple targets^7^. Both miR-19b and 720 and have involved in the development of vitiligo through CD8+ and CD4+ T cell response. CD8+ and CD4+ T cells in perilesional areas of non-segmental vitiligo exhibited a predominantly type-1-like cytokine profile, with secretion of IFN-γ and TNF-α^8^.

The primary purpose of this research is to analyze the levels of miRNAs expression (miR-16, 146a, 19b and 720) between vitiligo patients and healthy individuals. The results of this research aim in finding if miRNAs have a potential role in the pathogenesis and early diagnosis of vitiligo.

## Subjects and methods

Twenty vitiligo patients fulfilling the rules of the Declaration of Helsinki 1975 were enrolled in this study. The study protocol was reviewed and approved by Faculty of Medicine, Fayoum University, with approval number EC-395. All methods were performed in accordance with the relevant guidelines and regulations. A written informed consent was obtained from each subject before the start of the study as well. Another twenty healthy control studies were included as well. Vitiligo area scoring index (VASI)^9^ scores were calculated for all patients to assess the severity of the disease, and vitiligo disease activity (VIDA) scores calculated to assess disease activity^10^.

The inclusion criteria included adult subjects above 18 years old where all subjects are non-segmental vitiligo patients. Patients receiving immunosuppressive drugs; pregnant or lactating females; presence of other inflammatory or autoimmune diseases; and presence of leucoderma secondary to other causes were excluded from the study. Patients also undergoing treatment with immunosuppressive drugs or PUVA in the last 6 months were excluded.

### Molecular biology techniques

#### miR-16, miR-146a, miR-19b and miR-720 expressions in serum

From each subject, 5 mL were withdrawn in plain tube and centrifuged for 10 min at 4000×*g* to separate serum. Total RNA including miRNA was extracted from separated serum using (Qiagen, Valencia, CA, USA). RNA samples were subjected to RNA quantitation and purity assessment using the NanoDrop® (ND)-1000 spectrophotometer (NanoDrop Technologies, Inc., Wilmington, USA). Reverse transcription (RT) on total RNA was done in a final volume of 20 μL using the miScript II RT kit (Qiagen, Valencia, CA, USA). Quantitative Real-time PCR (qRT-PCR) for detection of miR-16, 146a, 19b and 720 took place.

Mature miRNA quantitative detection was done using miScript SYBER Green PCR kit (Qiagen, cat. no. 218073). Hs miRNA-16, Hs miRNA-146a, miRNA-19b and Hs miRNA-720 are the target-specific primers assay (forward primers) used for the selected miRNAs in addition to the housekeeping gene (internal control) Hs SNORD68, in this step.

Firstly, the stored cDNA samples, miScript SYBR Green PCR, and miScript Primer Assay kits were allowed to thaw at room temperature. Then, the reaction mix of a total volume of 25 μL was prepared by using the following components 12.5 μL QuantiTect SYBR Green PCR Master Mix (2×), 2.5 μL miScript Universal primer (10×), 2.5 μL miScript Primer assay (10×), 5 μL RNase free water and 2.5 μL Template cDNA.

Rotor-GeneQ72-well rotor (Qiagen,USA) was used as followed: incubation at 95 °C for 15 min for the initial activation step followed by 40 cycles each cycle was formed of 3 steps cycling of DNA denaturation at 94 °C for 15 s, annealing at 55 °C for 30 s and extension for 70 °C for 30 s.

Melting curves were analyzed after the completion of qRT-PCR cycles to validate and confirm the targeted miRNAs’ specific expression. Calculations of cycle threshold (Ct) values were calculated.

The relative expression of SNORD-68 was evaluated using the Ct method by the subtraction of Ct values of SNORD-68 from Ct values of the targeted miRNAs and this was done for both patients’ and control groups. Then, the calculation of Ct values was performed by subtracting Ct values of the control group from Ct values of the patients’ group. Finally, the fold changes (FC) which is the expression ratio or relative quantitation (Rq) for the target miRNAs were calculated using the 2^−ΔΔCt^ method.

#### miR-16, miR-146a, miR-19b and miR-720 expressions in tissue

For the tissue expression of miR-16, 146a, 19b and 720 RNA, same kit was used. 3 mm skin biopsy was taken from lesional regions from each subject and stored at − 80 °C until the time of analysis. At first, the area to be biopsied was cleaned by alcohol swab, anesthetize with local anesthetic as predocain and checked for numbness. At last, bandage biopsy site and care with local antibiotic. The tissue samples were homogenized with Qiazol lysis reagent that was provided with the RNA extraction kit. All the steps of RNA extraction, RT and qRT-PCR were the same like that of serum.

#### Statistical analysis of data

Organizing data and analysis were performed using statistical package of social science (SPSS 17.0) on windows 8.1. Arithmetic means and standard deviation were used. Test techniques used include, Independent Student *t*-test and One-way ANOVA test one for comparing 2-independent groups and the other for more than 2-independent groups, Kruskal–Wallis and Mann–Whitney test were used to compare more than 2-independent groups and to test the significance between groups. p-value < 0.05 was considered as a cutoff value for significance.

## Results

### Demographic and clinical characteristics of study population

In the present study 20 healthy controls (7 males, 13 females) with mean age 36.65 ± 11.636 years; 20 patients with vitiligo (8 males and 12 females) with mean age 36.85 ± 13.260 years were enrolled in this study. Demographic and clinical characteristics of vitiligo patients and controls are summarized in Table 1, no significant difference was observed in age and sex ratio among the two groups (p > 0.05).

**Table 1:** Demographic and clinical characteristics of study population.

Parameters	Control	Vitiligo	p-value
Age	36.65 ± 11.636	36.85 ± 13.26	0.45
Sex (N %)			
Female	13 (65%)	12 (60%)	0.75
Male	7 (35%)	8 (40%)	
Duration	–	78.55 ± 16.67	–
Extent (%)	–	18.12 ± 3.98	–
VASI	–	5.24 ± 1.80	–
VIDA	–	2.15 ± 1.53	–

Data is shown as mean-SD, p-values are statistically significant (p ≤ 0.05). *VASI* Vitiligo Area and Severity Index, *VIDA* Vitiligo Disease Activity Score. The Chi-square test is used for clinical manifestation variables.

### Expression levels of miR-16, miR-146a, miR-19b and miR-720 in serum and tissue for vitiligo and control groups

miR-16, miR-146a, miR-19b, miR-720 were examined in both serum and tissue among vitiligo group compared to healthy controls. Results revealed that in serum, expression levels for miR-16, 146a, 19b were overexpressed 3.90 ± 1.81, 3.65 ± 1.30 and 3.27 ± 1.65 for vitiligo patients as compared to healthy controls 1.06 ± 0.12; 1.06 ± 0.12 and 1.065 ± 0.1288 respectively with p-value(s) 0.000 for all biomarkers.

**Fig. 1:**
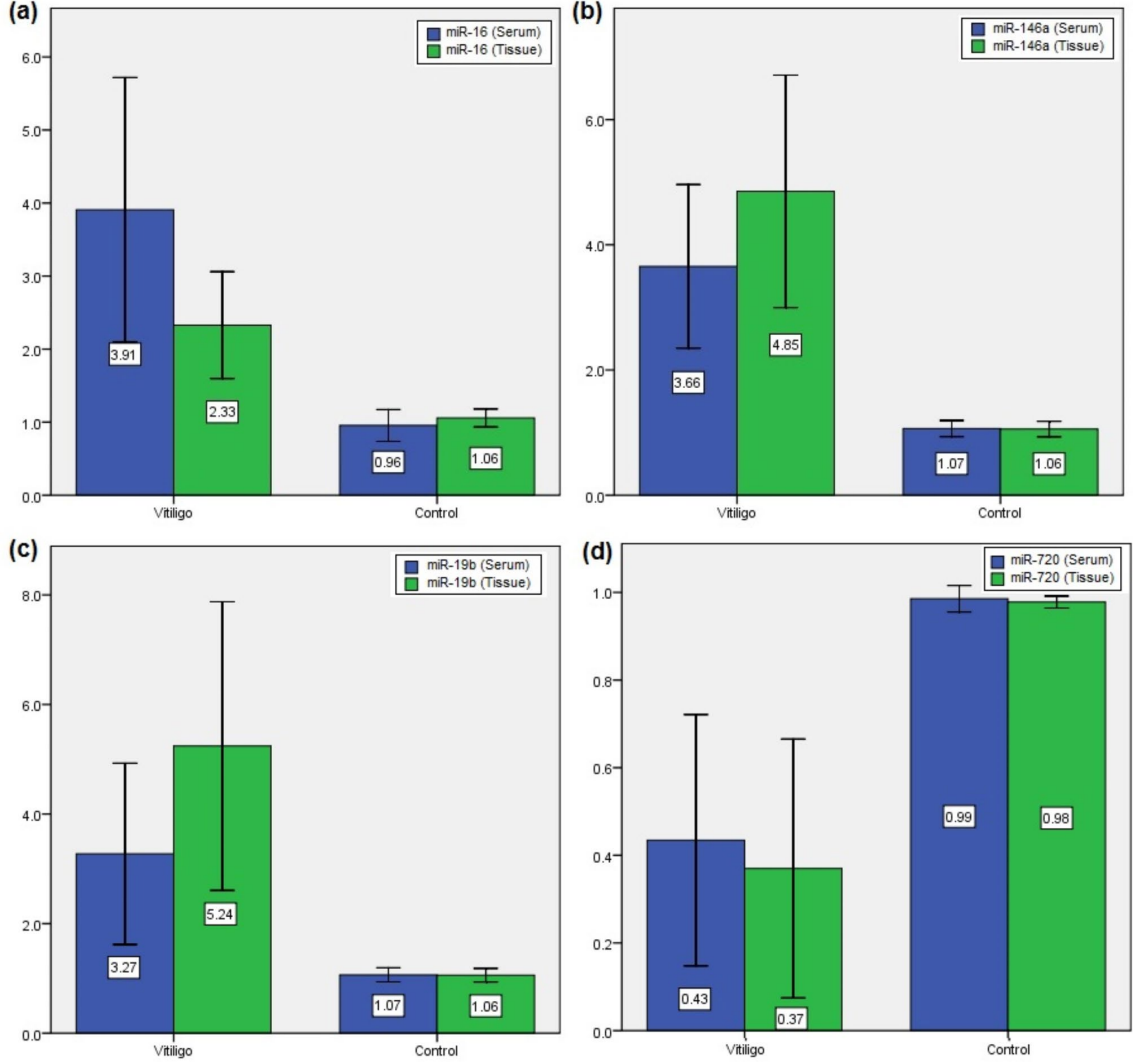
Expression levels (**a**) miR-16 serum vs tissue, (**b**) miR-146a serum vs tissue, (**c**) miR-19b serum vs tissue, (**d**) miR-720 serum vs tissue for vitiligo and control groups.

While miR-720 was reported low in vitiligo patients compared to control group 0.43 ± 0.28 and 0.98 ± 0.03 respectively with p-values 0.000. After evaluating the levels of the biomarkers in tissue, results revealed that miR-16, 146a, 19b were overexpressed in vitiligo patients 2.32 ± 0.73; 4.85 ± 1.857 and 5.24 ± 2.63 respectively and controls were 1.05 ± 0.12; 1.06 ± 0.124 and 1.05 ± 0.123 with p-values 0.000, 0.000 and < 0.001 respectively. While for the expression level of miR-720 in tissue, the level was low in vitiligo patients as compared to controls with mean ± SD of 0.37 ± 0.29 and 0.97 ± 0.013 respectively (p = 0.000) (Fig. 1).

### Correlations between biomarkers expression levels and clinical characteristics among patients

**Fig. 2:**
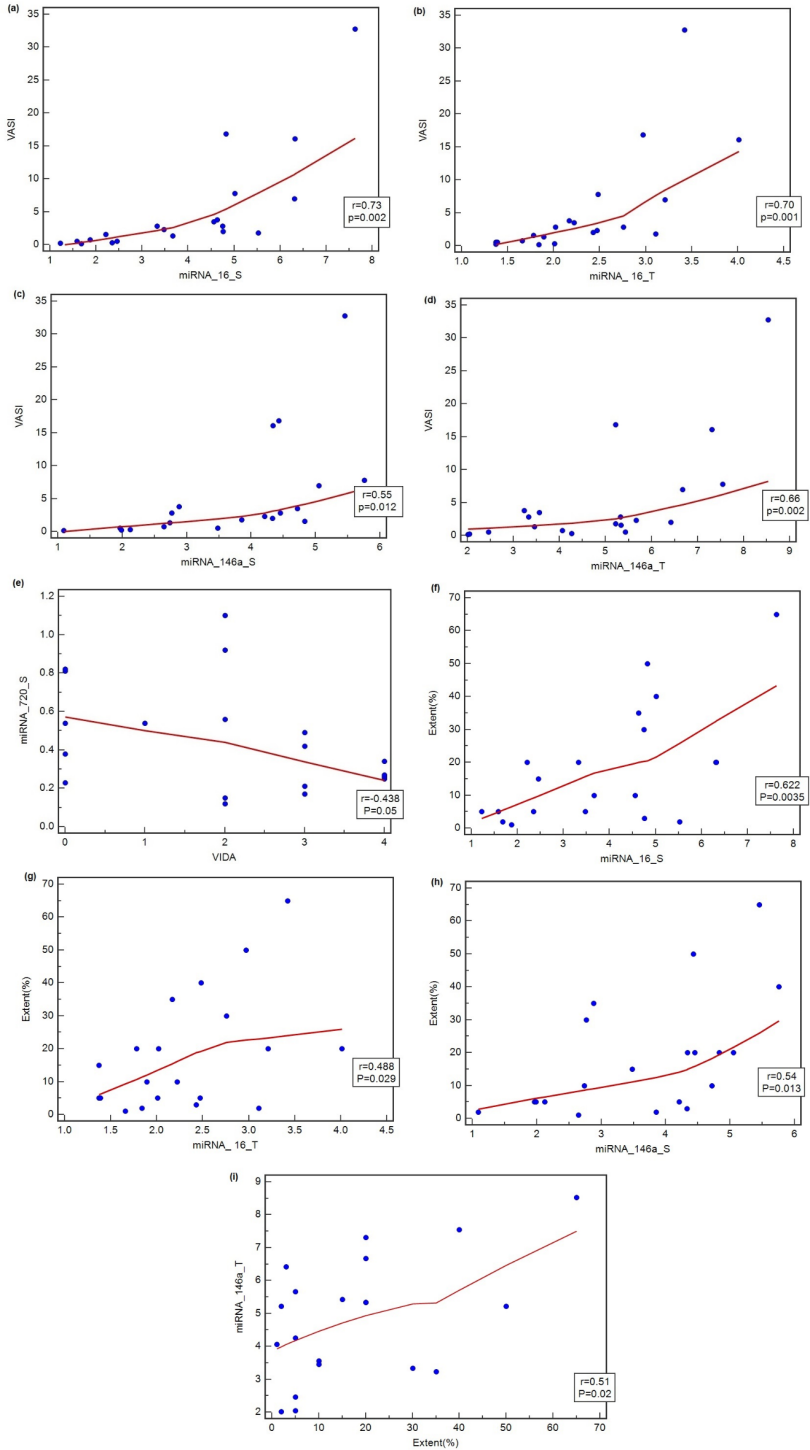
Correlations between (**a**) VASI and miR-16 in serum, (**b**) VASI and miR-16 in tissue, (**c**) VASI and miR-146a in serum, (**d**) VASI and miR-146a in tissue, (**e**) VIDA and miR-720 in serum, (**f**) extent of the disease and miR-16 in serum, (**g**) extent of the disease and miR-16 in tissue, (**h**) extent of the disease and miR-146a in serum, (**i**) extent of the disease and miR-146a in tissue.

After measuring the bivariate correlation using Pearson correlation coefficient, results show that there are positive correlations between miR-16 in serum and VASI (r = 0.73, p = 0.002), between miR-16 in tissue and VASI (r = 0.70, p = 0.001), between miR-146a in serum and VASI (r = 0.55, p = 0.012), between miR-146a in tissue and VASI (r = 0.66, p = 0.002). There are also positive correlations between extent percentage of vitiligo and miR-16 in serum and tissue (r = 0.622, p = 0.0035) and (r = 0.488, p = 0.029) respectively. Also we found positive correlations between the extent and miR-146a in both serum and tissue (r = 0.54, p = 0.013) and (r = 0.51, p = 0.02) respectively. There is also a negative correlation between miR-720 in serum and VIDA (r = − 0.438, p = 0.05) in vitiligo patients (Fig. 2). There are no correlations between serum and tissue biomarkers and between age/disease duration where p-value > 0.05. No correlation between extent percentage and miR-19b and 720 neither in serum nor tissue (p-value > 0.05).

Regarding gender, we reported that there is a statistical significant difference between VASI and gender with p-value 0.042, also between gender and miR-720 expression level in serum with p-value 0.025. There are no other significance between clinical, serum biomarkers and gender among vitiligo patients (Table 2).

**Table 2:** Relation between demographic, clinical characteristics, biomarkers as regards to gender in vitiligo patients. Significant values are in bold.

Parameters	Gender		p-value
	Male	Female	
Age	38.5 ± 13.62	35.75 ± 13.49	0.81
VASI	7.56 ± 11.36	3.69 ± 4.85	**0.042**
VIDA	1.75 ± 1.67	2.42 ± 1.44	0.72
miR-16 (serum)	4.47 ± 2.11	3.53 ± 1.56	0.48
miR-16 (tissue)	2.59 ± 0.87	2.15 ± 0.60	0.25
miR-146a (serum)	3.98 ± 1.09	3.43 ± 1.43	0.20
miR-146a (tissue)	5.47 ± 2.09	4.44 ± 1.65	0.71
miR-19b (serum)	3.76 ± 1.68	2.94 ± 1.63	0.42
miR-19b (tissue)	5.87 ± 2.59	4.82 ± 2.69	0.82
miR-720 (serum)	0.60 ± 0.31	0.32 ± 0.21	**0.025**
miR-720 (tissue)	0.46 ± 0.33	0.31 ± 0.27	0.67

### Correlations between expression levels of miR-16, 146a, 19b and 720 in serum and tissue among vitiligo patients

Our findings in this study prove that there are positive correlations between various biomarkers with p-value ≤ 0.05. Table 3 shows correlations between all biomarkers.

**Table 3:** Correlation between miR-16, 146a, 19b and 720 in serum and tissue among vitiligo patients. Significant values are in bold.

	miR-16 (Serum)	miR-16 (Tissue)	miR-146a (Serum)	miR-146a (Tissue)	miR-19b (Serum)	miR-19b (Tissue)	miR-720 (Serum)	miR-720 (Tissue)
miR-16 (Serum)	1.00	0.90	0.69	0.72	0.63	0.51	0.34	0.26
		**0.001**	**0.001**	**0.001**	**0.001**	**0.019**	0.15	0.28
miR-16 (Tissue)	0.90	1.00	0.57	0.67	0.55	0.34	0.23	0.09
	**0.001**		**0.009**	**0.001**	**0.01**	0.14	0.34	0.72
miR-146a (Serum)	0.69	0.57	1.00	0.85	0.65	0.67	0.28	0.01
	**0.001**	**0.009**		**p < 0.000**	**0.001**	**0.001**	0.23	0.97
miR-146a (Tissue)	0.72	0.673	0.85	1.00	0.77	0.76	0.38	− 0.12
	**0.001**	0.00	0.00		**0.000**	**0.001**	0.10	0.63
miR-19b (Serum)	0.63	0.559	0.65	0.77	1.00	0.89	0.57	0.19
	**0.001**	**0.01**	**0.001**	**0.001**		**p < 0.000**	**0.009**	0.43
miR-19b (Tissue)	0.52	0.34	0.67	0.76	0.89	1.00	0.64	0.14
	**0.02**	0.14	**0.001**	**0.001**	**0.001**		**0.001**	0.54
miR-720 (Serum)	0.34	0.23	0.28	0.38	0.57	0.64	1.00	0.43
	0.15	0.34	0.23	0.10	**0.009**	**0.001**		0.06
miR-720 (Tissue)	0.26	0.09	0.01	− 0.12	0.19	0.14	0.43	1.00
	0.28	0.72	0.97	0.63	0.43	0.54	0.06	

### ROC curve analysis for serum and tissue levels of miR-16, 146a, 19b and 720 in vitiligo patients

We performed ROC analysis to assess the diagnostic accuracy of serum and tissue biomarker levels of miR-16,146a, and 19b and 720 in detecting early-stage of vitiligo. Accordingly we conducted a retrospective power calculation using the observed AUC of (0.90–0.98) and with the sample size (n = 20), the power of our analysis was estimated to be 0.75. The 95% confidence intervals around the AUC have also been reported to reflect the precision of the estimates. However this is slightly lower than the commonly accepted threshold of 0.8, the bootstrapping technique and confidence intervals we applied provide further robustness to the analysis. Previous study proved that with a limited sample size of 20 non-segmental vitiligo patients and applying ROC analysis, it could be acceptable^11^.

**Fig. 3:**
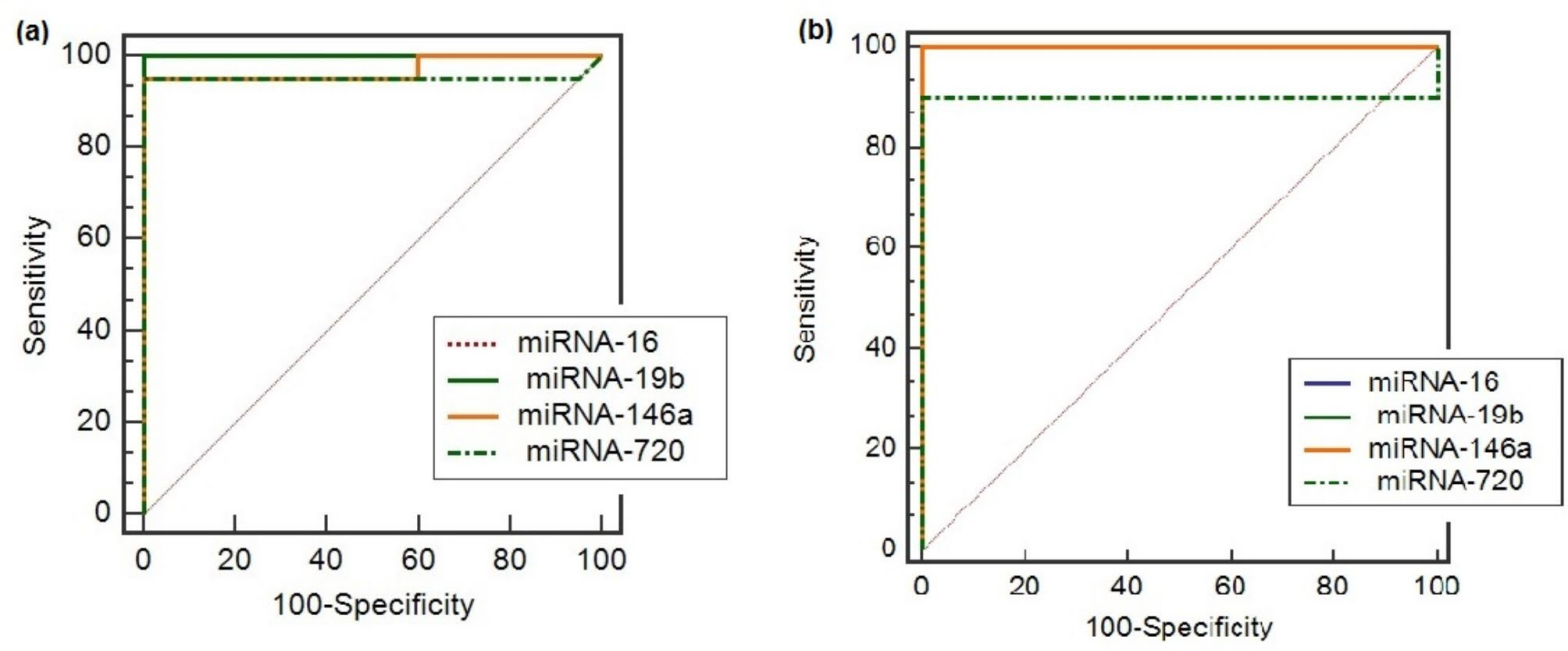
ROC curve analysis for (**a**) miR-16, 146a, 19b and 720 levels in serum, (**b**) levels of miR-16, 146a, 19b and 720 levels in tissue among vitiligo patients.

According to the pairwise comparison of ROC curves in serum level, results revealed that the 95% CI between miR-16_miR-19b (0.0001–0.0002) p > 0.05; between miR-16_miR-146a (− 0.030 to 0.090) p = 0.32; between miR-16_miR-720 (− 0.047 to 0.144) p = 0.31; between miR-19b_miR-146a (− 0.0298 to 0.0898) p = 0.32; between miR-19b_miR-720 (− 0.047 to 0.144) p = 0.31 and between miR-146a_miR-720 (− 0.097 to 0.134) p = 0.75 (Fig. 3a).

For tissue level, results revealed that the 95% CI between miR-16_miR-19b (0.0001 to 0.0002) p > 0.05; between miR-16_miR-146a 0.0001 to 0.0002) p > 0.05; between miR-16_miR-720 (− 0.035 to 0.235) p = 0.146; between miR-19b_miR-146a (0.0001 to 0.0002) p > 0.05; between miR-19b_miR-720 (− 0.035 to 0.235) p = 0.146 and between miR-146a_miR-720 (− 0.035 to 0.235) p = 0.146 (Fig. 3b).

## Discussion

The pathogenesis of vitiligo is still unclear as general. Based on a growing number of studies, miRNAs showed a vital role in autoimmune disorders. They have been shown to be unusually well preserved in serum or plasma derived from immune cells and other tissues and serve as promising biomarkers for different diseases including non-segmental vitiligo^12^.

In this study, we detected that the expression levels for miR-16, 146a, 19b were overexpressed in the both serum and tissue of vitiligo patients compared to healthy controls. While for the expression level of miR-720, our findings concluded that it was reported low in vitiligo patients compared to control group (serum and tissue).

As agreement to our results, a study on 10 non-segmental vitiligo patients and 20 healthy controls by microRNA arrays showed significantly different expression levels between the two groups. The expression levels of miR-16 and miR-19b were upregulated, and miR-720 was downregulated in the serum. They showed that miR-16, 19b and 720 are considered as potential biomarkers for vitiligo^13^.

Also, some authors^14–16^ are in agreement with current study as they reported significant upregulation of miRNA-146a expression in patients with vitiligo in comparison with healthy controls and they concluded that miRNA-146a became a part of the vitiligo-associated immune response.

Another study that was performed on serum miRNA expressions profiles in 10 patients with NSV and 20 healthy controls by microRNA arrays. The study found that miRNA 19b showed significantly different expression levels between the two groups^17^. Also, recently^18^ determined by RNA-seq in their finding that hsa-miR-19b-3p expression was high in progressive non-segmental vitiligo compared to healthy controls and that there is a statistically significant between both groups.

Many studies reported the importance of miRNAs in the pathogenesis of vitiligo^2^. Another studies reported that as for the Melanocyte function in vitiligo, miR-720 plays an important role where for non-segmental vitiligo patients, miR-720 is downregulated in serum showing significantly different expression levels between the two groups (vitiligo patients and healthy controls)^19^.

On the contrary to our results, another study investigated the expression and potential role of miRNAs in vitiligo where authors proved that miR-99b, miR-125b, miR-155 and miR-199a-3p levels were found to be increased while that of miR-145 was found to be decreased in the skin of patients with vitiligo. Although they reported no significant overlap between miR-145 targets and genes associated with pigmentation, they detected the presence of binding sites for miR-145 in the mRNAs of melanogenesis-associated genes^20^.

Also disagreeing to our finding, Šahmatova et al. could not detect any significant difference in the relative levels of miRNA-146a expressions between vitiligo lesions and normal control skin. This discrepancy could be attributed to the fact that they assessed miRNA-146a expression in skin lesions not in the blood^20^.

In the present study as well, we found positive correlations between VASI and miR-16 in serum and between VASI and miR-16 in tissue. Also, positive correlation between VASI and miR-146a in serum and in tissue as well. We reported positive correlations between extent percentage of vitiligo and miR-16 in serum and in tissue as well; between the extent and miR-146a in both serum and tissue. Negative correlation between miR-720 in serum and VIDA was reported as well. We reported no correlations between any biomarkers either in serum or in tissue and between age/disease duration. No correlation between extent percentage and miR-19b and 720 neither in serum nor tissue.

On the contrary, a study proved that there is a positive role of miRNA-146a in the pathogenesis of immune-mediated diseases, authors could not detect any significant correlation between levels of miRNA-146a in patients with vitiligo and their clinical parameters except for VIDA score of disease activity^16^.

As previously mentioned in the results, our findings in this study showed that there are mixed positive correlations between biomarkers in serum and in tissue.

To the best of our knowledge this is the first study to have that mixed combination of miRNAs evaluated in both serum and tissue of vitiligo patients. The correlations we have made can be used as an early predictable diagnosis design for vitiligo.

## Conclusions

In conclusion, the upregulated expression of miR-16, 146a, 19b in serum and tissue; as well as the downregulation expression of miR-720 in serum and tissue are correlated with the pathogenesis of vitiligo. These biomarkers can play a positive role in the early diagnosis of non-segmental vitiligo.

## Data Availability

The data that support the findings of this study are not included in the manuscript for ethical reasons but are available upon reasonable request from the corresponding author, Olfat Shaker [email: olfat.shaker@kasralainy.edu.eg].

## References

[1] Czajkowski, R. & Męcińska-Jundziłł, K. Current aspects of vitiligo genetics. *Postepy dermatologii i alergologii* **31**(4), 247–255 (2014). 25254010 10.5114/pdia.2014.43497 PMC4171675

[2] Raia, N., Shaker, O. G., Hassan, Z. M. & Abd Elrahim, T. A. Is there a relation between long non-coding RNA MALAT-1 and miRNA-9 in Egyptian patients with Vitiligo?. *Exp. Dermatol.* **31**(3), 381–383 (2022). 34714557 10.1111/exd.14487

[3] Rebane, A. & Akdis, C. A. MicroRNAs: Essential players in the regulation of inflammation. *J. Allergy Clin. Immunol.* **132**(1), 15–26 (2013). 23726263 10.1016/j.jaci.2013.04.011

[4] Lee, J. S. et al. MicroRNA-365a/b-3p as a potential biomarker for hypertrophic scars. *Int. J. Mol. Sci.* **23**(11), 6117 (2022). 35682793 10.3390/ijms23116117 PMC9181131

[5] Zhang, Z. et al. Differentially expressed microRNAs in peripheral blood mononuclear cells of non-segmental vitiligo and their clinical significance. *J. Clin. Lab. Anal.* **35**(2), e23648 (2021). 33169883 10.1002/jcla.23648 PMC7891539

[6] Lu, Z. et al. MiR-15a/16-1 suppresses AHR-dependent IL-22 secretion in CD4+ T cells and contributes to immune-mediated organ injury. *Hepatology* **67**(3), 1027–1040 (2018). 29023933 10.1002/hep.29573

[7] Hermann, H. et al. miR-146b probably assists miRNA-146a in the suppression of keratinocyte proliferation and inflammatory responses in psoriasis. *J. Investig. Dermatol.* **137**(9), 1945–1954 (2017). 28595995 10.1016/j.jid.2017.05.012 PMC5977389

[8] Riding, R. L. & Harris, J. E. The role of memory CD8+ T cells in vitiligo. *J. Immunol.* **203**(1), 11–19 (2019). 31209143 10.4049/jimmunol.1900027 PMC6709670

[9] Alghamdi, K. M., Kumar, A., Taïeb, A. & Ezzedine, K. Assessment methods for the evaluation of vitiligo. *J. Eur. Acad. Dermatol. Venereol. JEADV* **26**(12), 1463–1471 (2012). 22416879 10.1111/j.1468-3083.2012.04505.x

[10] Dicle, O. Assessment methods in vitiligo. *Pigment. Disord.* **2**, 160 (2015).

[11] Moftah, N. H., Alnos, H., Rashed, L. & Hamdino, M. Evaluation of serum and tissue levels of cold-inducible RNA-binding protein in non-segmental Vitiligo. *Arch. Dermatol. Res.* **315**(7), 2065–2071 (2023). 36920542 10.1007/s00403-023-02586-6 PMC10366246

[12] Hulstaert, E. et al. Charting extracellular transcriptomes in the human biofluid RNA atlas. *Cell Rep.* **33**(13), 108552 (2020). 33378673 10.1016/j.celrep.2020.108552

[13] Shi, Y. L. et al. MicroRNA expression profiling identifies potential serum biomarkers for non-segmental vitiligo. *Pigment Cell Melanoma Res.* **26**, 418–421 (2013). 23470042 10.1111/pcmr.12086

[14] Shi, Y. L., Weiland, M., Lim, H. W., Mi, Q. S. & Zhou, L. Serum miRNA expression profiles change in autoimmune vitiligo in mice. *Exp. Dermatol.* **23**(2), 140–142 (2014). 24401108 10.1111/exd.12319

[15] Wang, P. et al. The changes of microRNA expression profiles and tyrosinase related proteins in MITF knocked down melanocytes. *Mol. Biosyst.* **8**, 2924–2931 (2012). 22898827 10.1039/c2mb25228g

[16] Hassan, A. M., Neinaa, Y. E., El-Bendary, A. S. & Zakaria, S. S. MicroRNA-146a and Forkhead box protein 3 expressions in nonsegmental vitiligo: an insight into disease pathogenesis. *J. Egypt. Womens Dermatol. Soc.* **16**, 105–111 (2019).

[17] Issa, Y. W. & Salih, S. M. Impact of miR-155, miR-145 and miR-328 on pigmentary process in Iraqi patients with vitiligo. *Gene Rep.* **21**, 100955 (2020).

[18] de França, E. et al. Potential role of chronic physical exercise as a treatment in the development of vitiligo. *Front. Physiol.*10.3389/fphys.2022.843784 (2022). 35360245 10.3389/fphys.2022.843784 PMC8960951

[19] Li, L. The role of microRNAs in vitiligo: regulators and therapeutic targets. *Ann. Dermatol.* **32**(6), 441–451 (2020). 33911786 10.5021/ad.2020.32.6.441 PMC7875238

[20] Šahmatova, L. et al. MicroRNA-155 is dysregulated in the skin of patients with vitiligo and inhibits melanogenesis-associated genes in melanocytes and keratinocytes. *Acta Dermato-venereol.* **96**(6), 742–748 (2016). 10.2340/00015555-2394 26941046

